# Apple Flavonols Mitigate Adipocyte Inflammation and Promote Angiogenic Factors in LPS- and Cobalt Chloride-Stimulated Adipocytes, in Part by a Peroxisome Proliferator-Activated Receptor-γ-Dependent Mechanism

**DOI:** 10.3390/nu12051386

**Published:** 2020-05-12

**Authors:** Danyelle M. Liddle, Meaghan E. Kavanagh, Amanda J. Wright, Lindsay E. Robinson

**Affiliations:** Department of Human Health and Nutritional Sciences, University of Guelph, Guelph, ON N1G 2W1, Canada; dliddle@uoguelph.ca (D.M.L.); kavanagh.meaghan.e@gmail.com (M.E.K.); ajwright@uoguelph.ca (A.J.W.)

**Keywords:** adipocyte, flavonols, hypoxia, inflammation, obesity

## Abstract

Adipose tissue (AT) expansion induces local hypoxia, a key contributor to the chronic low-grade inflammation that drives obesity-associated disease. Apple flavonols phloretin (PT) and phlorizin (PZ) are suggested anti-inflammatory molecules but their effectiveness in obese AT is inadequately understood. Using in vitro models designed to reproduce the obese AT microenvironment, 3T3-L1 adipocytes were cultured for 24 h with PT or PZ (100 μM) concurrent with the inflammatory stimulus lipopolysaccharide (LPS; 10 ng/mL) and/or the hypoxia mimetic cobalt chloride (CoCl_2_; 100 μM). Within each condition, PT was more potent than PZ and its effects were partially mediated by peroxisome proliferator-activated receptor (PPAR)-γ (*p* < 0.05), as tested using the PPAR-γ antagonist bisphenol A diglycidyl ether (BADGE). In LPS-, CoCl_2_-, or LPS + CoCl_2_-stimulated adipocytes, PT reduced mRNA expression and/or secreted protein levels of inflammatory and macrophage chemotactic adipokines, and increased that of anti-inflammatory and angiogenic adipokines, which was consistent with reduced mRNA expression of M1 polarization markers and increased M2 markers in RAW 264.7 macrophages cultured in media collected from LPS + CoCl_2_-simulated adipocytes (*p* < 0.05). Further, within LPS + CoCl_2_-stimulated adipocytes, PT reduced reactive oxygen species accumulation, nuclear factor-κB activation, and apoptotic protein expression (*p* < 0.05). Overall, apple flavonols attenuate critical aspects of the obese AT phenotype.

## 1. Introduction

In diet-induced obesity, adipose tissue (AT) expansion is accompanied by metabolic dysfunction [1]. Several mechanisms have been proposed to explain the pathogenesis of obese AT dysfunction, including dysregulated adipocyte production of inflammatory adipokines (e.g., interleukin (IL)-1β and IL-6, monocyte chemoattractant protein (MCP)-1, tumor necrosis factor (TNF)-α) [2] via activation of toll-like receptor 4 (TLR4) by dietary saturated fatty acids (SFA) [3], or dysbiotic gut-derived endotoxin (i.e., lipopolysaccharide (LPS)) [4], the induction of metabolic stress [5] and increased activities of reactive oxygen species (ROS) [6], and the activation and consequent paracrine interactions with specific immune cell populations, particularly M1 macrophages [7]. A common intermediate amongst these mechanisms is activation of the nuclear factor kappa-light-chain-enhancer of activated B cells (NF-κB) transcription factor that regulates inflammatory adipokine expression [1,8]. Indeed, obesity is recognized as a chronic low-grade inflammatory state that drives the development of insulin resistance, a precursor to metabolic diseases such as type 2 diabetes (T2D) and cardiovascular disease (CVD) [8].

Another causative factor underlying obese AT inflammation is hypoxia [9]. When hypertrophic adipocyte size exceeds the diffusional limit of oxygen from the blood [10], the low oxygen tension induces both NF-κB and hypoxia-inducible factor (HIF)-1 accumulation [11,12,13]. As a transcription factor, HIF-1 regulates the adipocyte molecular response to hypoxia, mainly promoting gene expression of angiogenic adipokines (e.g., vascular endothelial growth factors (VEGF), angiopoietin-like peptides (Angptl), and leptin) to form new blood vessels [11,12]. However, in obese AT, the rate of angiogenesis is not sufficient to supply hypertrophic adipocytes with enough oxygen and nutrients [14], eventually leading to adipocyte apoptosis [15]. In response, certain immune cells (particularly macrophages) are recruited to AT to remove the cellular debris [16]; however, the increased and sustained immune cell infiltration and paracrine signaling with adipocytes drives local AT inflammation, leading to its dysfunction [17]. In this context, hypoxia may be an early event responsible for the initiation of AT inflammation and dysfunction, and strategies aimed at mitigating this sequence of events are warranted.

One possible dietary strategy is the intake of flavonoids, a group of plant-derived phenolic compounds associated with a reduced risk of metabolic diseases [18]. Of all fruit and vegetable flavonoid sources, apples are reportedly the most consumed in the Western diet [19] and have been consistently associated with a reduced risk of T2D and CVD [20]. Phlorizin (PZ), yielding the aglycone phloretin (PT), is the major dihydrochalcone flavonoid found in apples, primarily in the peel [21]. PT has been shown to promote adipogenic mRNA transcription via the peroxisome proliferator-activated receptor (PPAR)-γ transcription factor which also regulates the transcription of the anti-inflammatory adipokine adiponectin [22] and angiogenic adipokines [23]. Indeed, both PT and PZ have been shown to exert anti-inflammatory and anti-oxidant activities in vitro [22,24,25,26,27] and in vivo [28,29], but these effects and the underlying mechanisms have not been studied in the context of the obese AT inflammatory and hypoxic microenvironment. Thus, the purpose of this study was to investigate the effects of PT and PZ in modulating the adipocyte response to inflammatory and hypoxic stimuli, as well as their consequent effects on macrophage polarization, and to discern the role of PPAR-γ in mediating those responses.

## 2. Materials and Methods

### 2.1. Cell Culture and Differentiation

3T3-L1 murine pre-adipocytes (American Type Culture Collection (ATCC), USA) were cultured and differentiated according to the manufacturer’s instructions, as previously described [30]. Adipocytes were treated as described below eight days after differentiation was induced, when complete differentiation had occurred.

### 2.2. Treatments

All treatments were performed in triplicate and replicated independently three times (i.e., *n* = 9/treatment). All cells were treated for 24 h with 100 μM of the apple flavonols PT or PZ (from apple wood, ≥99% pure; Sigma–Aldrich, St. Louis, MO, USA) in combination with 10 ng/mL of the inflammatory stimulus LPS (from *Escherichia coli* 055:B5; Sigma–Aldrich) or 100 μM of the hypoxia mimetic cobalt chloride (CoCl_2_; Sigma–Aldrich) [11,12], or in combination with both LPS + CoCl_2_. PT and PZ were dissolved in lab-grade ethanol and LPS was dissolved in fetal bovine serum-free media. For PT and PZ, 100 μM was chosen as the optimal dose shown to modulate adipocyte gene expression when in co-culture with macrophages, without affecting cell viability [26]. Further, 100 μM PT is reportedly representative of the maximum concentration of total PT that was achieved in the plasma of PT- and PZ-red rodents [31]. We chose 10 ng/mL of LPS to mimic circulating endotoxin levels in obese humans [32] and rodents [4]. For CoCl_2_, we chose 100 μM to mimic the effects of low (1%) oxygen tension in adipocytes over 24 h without affecting cell viability [12]. Unstimulated adipocytes served as the negative control for each culture condition. To investigate the mechanism by which PT and PZ exert their effects, a PPAR-γ antagonist, bisphenol A diglycidyl ether (BADGE; Cayman Chemical, Ann Arbor, MI, USA) was added to a subset of each culture condition. BADGE was diluted in lab-grade ethanol and added to culture wells at 100 μM, as described [33]. Cell viability, assessed by trypan blue exclusion, exceeded 90% for all culture conditions, which was supported by no differences in total cellular protein content compared to unstimulated adipocytes. After 24 h, supernatant samples were collected, and cells were lysed to isolate RNA and protein using the RNA/Protein Purification Kit as per the manufacturer’s instructions (Norgen Biotek Corp., Thorold, ON, Canada).

### 2.3. RAW 264.7 Macrophages Treated with LPS + CoCl_2_-Stimulated Culture Conditioned Media

RAW 264.7 macrophages (ATCC) were maintained according to the manufacturer’s instructions, as previously described [34]. Macrophages were split, centrifuged and resuspended in fresh media 4 h prior to treatment with conditioned media from LPS + CoCl_2_-stimulated cultures ± BADGE (*n* = 9/treatment). Cell viability, assessed by trypan blue exclusion, exceeded 95%. We seeded 1 × 10^5^ macrophages into 96-well plates and allowed them to adhere before being cultured in conditioned media. After 6 h, supernatant samples were collected, and macrophages were lysed to isolate RNA.

### 2.4. mRNA and Secreted Protein Analyses

We used 2 μg of isolated RNA from all adipocyte and macrophage cultures to synthesize cDNA using a high-capacity cDNA reverse transcription kit (Applied Biosystems, Foster City, CA, USA) and real time polymerase chain reaction was performed using the 7900HT Fast Real Time PCR system (Applied Biosystems), as described [34]. Target gene expression was normalized to expression of the housekeeping gene, ribosomal protein, large, P0 (*Rplp0*), and the relative differences in gene expression between groups were determined using the ∆∆Ct method in comparison to unstimulated adipocytes. Primer sequences for inflammatory (*Il1β*, *Il6*, *Mcp1*, *Tnfα*), anti-inflammatory (*adiponectin*, *Il10*), and angiogenic (*Angptl4*, *leptin*, *Vegfa*) adipokines, additional M1 (cluster of differentiation (*Cd)11b*, *Cd11c*, inducible nitric oxide synthase (*iNos)*) and M2 (*Cd206*, transforming growth factor *(Tgf)β*) macrophage polarization markers, and related transcription factors (*Hif1α, Nfκb, Pparγ*) are shown in the Supplemental Materials Table S1. All reported fold-changes are relative to unstimulated adipocytes.

### 2.5. Secreted Protein Analysis

Secreted protein concentrations of inflammatory (IL-1β, IL-6, TNF-α), anti-inflammatory (adiponectin, IL-10), macrophage chemotactic (MCP-1, MCP-3, macrophage inflammatory protein (MIP)-1α, MIP-1β), and angiogenic (Angptl4, leptin, VEGF-A) adipokines were measured in supernatant samples collected at 24 h from LPS + CoCl_2_-stimulated cultures using a Mouse Cytokine/Chemokine Bio-Plex Pro kit (Bio-Rad, Mississauga, ON, Canada) and analyzed using the Bio-Plex 200 system/Bio-Plex Manager software Version 6.0 (Bio-Rad).

### 2.6. ROS Accumulation

ROS was detected in LPS + CoCl_2_-stimulated cultures (*n* = 9/treatment) at 24 h by a nitro blue tetrazolium (Sigma–Aldrich) assay, as previously described [30].

### 2.7. NF-κB p65 Activation

Total cellular protein was quantified using the bicinchoninic assay according to the manufacturer’s instructions (Fisher Scientific, Mississauga, ON, Canada) and an equal amount of protein (10 μg)/sample was utilized. NF-κB p65 activation was determined in LPS + CoCl_2_-stimulated cultures from the ratio of phosphorylated (i.e., activated) NF-κB p65 (Ser536) to total NF-κB p65 by enzyme-linked immunosorbent assay as per the manufacturer’s instructions (eBioscience, San Diego, CA, USA).

### 2.8. Cellular Apoptotic Protein Analysis

Cellular levels of the B-cell lymphoma 2 (BCL-2) protein family including apoptotic (BCL-2-associated agonist of cell death (BAD), BCL-2-like protein 11 (BIM)) and anti-apoptotic (BCL-2/BCL-2-like protein 4 (BAX), B-cell lymphoma-extra-large (BCL-XL), induced myeloid leukemia cell differentiation protein (MCL-1)) proteins were measured in LPS + CoCl_2_-stimulated cultures at 24 h using a Mouse Bio-Plex Pro Apoptosis kit (Bio-Rad) and analyzed using the Bio-Plex 200 system/Bio-Plex Manager software, Version 6.0 (Bio-Rad).

### 2.9. Statistical Analysis

All statistical analyses were performed using the Statistical Analysis System, University Edition (SAS Institute Inc., Cary, NC, USA) with *p* < 0.05 considered statistically significant. Data collected from LPS, CoCl_2_, and LPS + CoCl_2_-stimulated adipocytes were compared to data collected from unstimulated adipocytes using unpaired *t*-tests to confirm the adipocyte inflammatory and/or hypoxic response in each model (data not shown). Data collected from each culture condition were analyzed using a two-way ANOVA for the main effects of flavonol and BADGE treatment. The Shapiro–Wilk test confirmed normality for all endpoints and the Tukey post-hoc test was used to identify significant differences between groups. Replicate experiments were averaged and expressed as means ± standard error of the mean (SEM).

## 3. Results

### 3.1. PT and PZ Modulate Adipokine mRNA Expression in LPS-Stimulated Adipocytes, in Part by a PPAR-γ-Dependent Mechanism

To determine the effects of PT and PZ in modulating the adipocyte response to obese AT inflammation, PT- and PZ-treated adipocytes were co-stimulated for 24 h with a physiological dose of LPS. Compared to LPS-stimulated adipocytes, PT reduced mRNA expression of *leptin*, *Il1β*, *Il6* and *Mcp1*, while PZ less potently reduced only *Il6* and *Mcp1* (*p* < 0.05; Table 1). Both PT and PZ increased *adiponectin* mRNA expression (*p* < 0.05; Table 1) with PT being more potent, while neither affected *Vegfa*, *Angptl4*, *Tnfα*, *Il10*, *Hif1α*, *Nfκb*, or *Pparγ* mRNA levels (*p* > 0.05; Table 1). Interestingly, in LPS-stimulated adipocytes, PPAR-γ antagonism with BADGE further increased *leptin* expression (*p* < 0.05; Table 1) but did not affect any other analytes (*p* > 0.05; Table 1). The anti-inflammatory effects of PT and PZ were completely reversed by BADGE (*p* < 0.05; Table 1) with the exception of *Il1β* in PT-treated adipocytes wherein BADGE only partially reversed this anti-inflammatory effect (*p* < 0.05; Table 1). Collectively, these data highlight the anti-inflammatory effects of PT and PZ as well as the superior effectiveness of PT in adipocytes, and point to the central role of PPAR-γ in mediating these effects.

### 3.2. PT and PZ Modulate Adipokine mRNA Expression in CoCl_2_-Stimulated Adipocytes, in Part by a PPAR-γ-Dependent Mechanism

To determine the effects of PT and PZ in modulating the adipocyte response to obese AT hypoxia, PT- and PZ-treated adipocytes were co-stimulated for 24 h with the hypoxia mimetic CoCl_2_. In general, compared to LPS-stimulated cultures, CoCl_2_ increased the adipocyte angiogenic and inflammatory response *(p* < 0.05; Table 1; Table 2). Within CoCl_2_-stimulated cultures and compared to CoCl_2_-stimulation alone, PT reduced mRNA expression of *leptin*, *Il1β*, *Il6*, *Mcp1*, and *Nfκb*, and increased that of *Vegfa*, *Angptl4*, *adiponectin*, and *Pparγ* (*p* < 0.05; Table 2), with no effect on *Tnfα*, *Il10*, or *Hif1α* (*p* > 0.05; Table 2). Likewise, PZ reduced mRNA expression of *leptin*, *Il1β*, *Il6*, and *Mcp1*, and increased *Vegfa* and *adiponectin* (*p* < 0.05; Table 2), but with less potency compared to PT (*p* < 0.05; Table 2). PZ did not affect *Angptl4*, *Tnfα*, *Il10*, *Hif1α*, *Nfκb*, or *Pparγ* mRNA levels (*p* > 0.05; Table 2). Interestingly, PPAR-γ inhibition with BADGE further increased *leptin* and *Nfκb* mRNA expression in CoCl_2_-stimulated adipocytes (*p* < 0.05; Table 2) but did not affect any other analytes (*p* > 0.05; Table 2). The angiogenic and anti-inflammatory effects of PT on *leptin*, *adiponectin*, *Nfκb*, and *Pparγ* mRNA expression were totally reversed by BADGE (*p* < 0.05; Table 2), whereas the effects on *Vegfa*, *Angptl4*, *Il1β*, *Il6*, and *Mcp1* were only partially reversed (*p* < 0.05; Table 2). Finally, the effects of PZ were totally reversed by BADGE with the exception of *leptin* mRNA expression, which was only partially reversed (*p* < 0.05; Table 2). Collectively, these data highlight the anti-inflammatory and angiogenic effects of PT and PZ in adipocytes under hypoxic conditions, as well as the superior effectiveness of PT. Further, these data point to the central role of PPAR-γ in mediating the effects of PZ and suggest an additional, PPAR-γ-independent mechanism through which PT exerts its anti-inflammatory and angiogenic effects in hypoxic adipocytes.

### 3.3. PT but not PZ Modulates Adipokine mRNA Expression in LPS + CoCl_2_-Stimulated Adipocytes, in Part by a PPAR-γ-Dependent Mechanism

**Figure 1:**
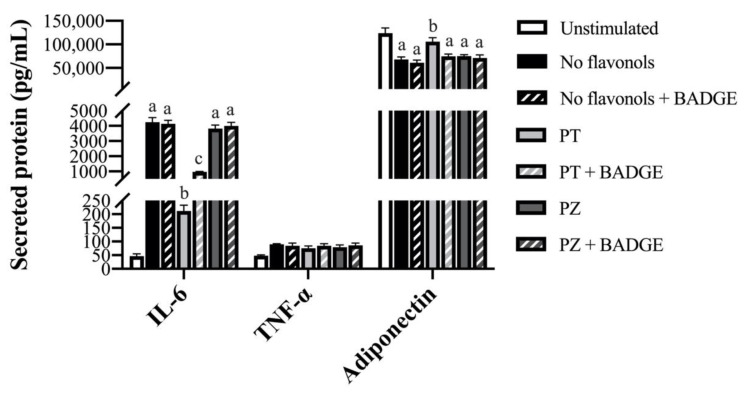
Secreted protein levels of inflammatory (interleukin (IL)-6 and tumor necrosis factor (TNF)-α) and anti-inflammatory (adiponectin) adipokines in lipopolysaccharide (LPS) + cobalt chloride (CoCl_2_)-stimulated adipocytes co-treated with phloretin (PT) or phlorizin (PZ), with or without bisphenol A diglycidyl ether (BADGE). Values are means ± standard error of the mean (SEM); *n* = 9/treatment. Data were analyzed by two-way ANOVA and means without a common letter differ within cultures treated ± BADGE, *p* < 0.05.

**Figure 2:**
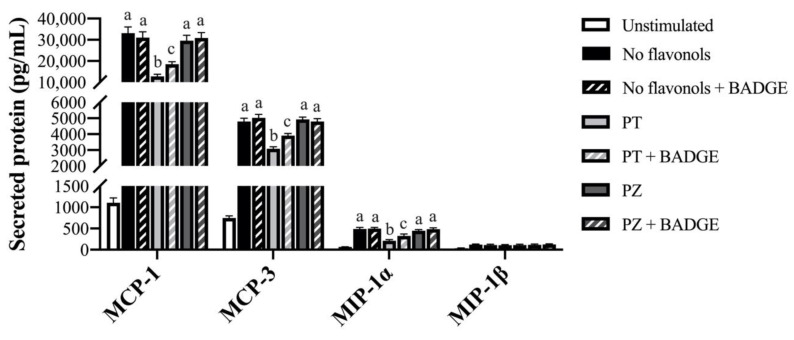
Secreted protein levels of macrophage chemotactic adipokines (monocyte chemoattractant protein (MCP)-1, MCP-3, macrophage inflammatory protein (MIP)-1α and MIP-1β) in LPS + CoCl_2_-stimulated adipocytes co-treated with PT or PZ, with or without BADGE. Values are means ± SEM; *n* = 9/treatment. Data were analyzed by two-way ANOVA and means without a common letter differ within cultures treated ± BADGE, *p* < 0.05.

**Figure 3:**
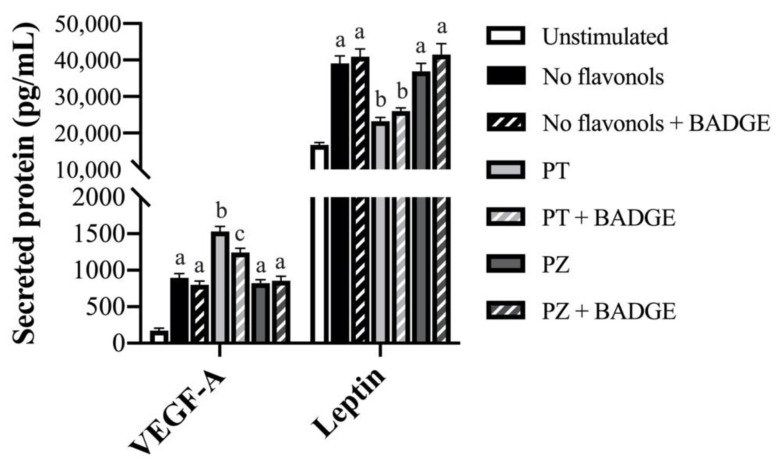
Secreted protein levels of angiogenic factors (vascular endothelial growth factor (VEGF)-A and leptin) in LPS + CoCl_2_-stimulated adipocytes co-treated with PT or PZ, with or without BADGE. Values are means ± SEM; *n* = 9/treatment. Data were analyzed by two-way ANOVA and means without a common letter differ within cultures treated ± BADGE, *p* < 0.05.

To determine the effects of PT and PZ in modulating the adipocyte response to a combined inflammatory and hypoxic microenvironment which best represents obese AT, PT- and PZ-treated adipocytes were co-stimulated for 24 h with a physiological dose of LPS and CoCl_2_. In general, compared to separate LPS- and CoCl_2_-stimulated cultures, combined LPS + CoCl_2_ stimulation decreased the adipocyte angiogenic response and increased the inflammatory response (*p* < 0.05; Table 1, Table 2 and Table 3), thus confirming this model as best representative of the obese AT adipocyte phenotype [1]. Importantly, PPAR-γ antagonism with BADGE did not affect LPS + CoCl_2_-stimulated adipocyte mRNA expression (Table 3) or secreted adipokine levels (*p* > 0.05; Figure 1, Figure 2 and Figure 3). Within LPS + CoCl_2_-stimulated cultures and compared to LPS + CoCl_2_ stimulation alone, PT reduced mRNA expression of *leptin*, *Il1β*, *Il6*, *Mcp1*, *Tnfα*, *Hif1α*, and *Nfκb*, and increased *Vegfa*, *Angptl4*, *adiponectin*, and *Pparγ* (*p* < 0.05; Table 3), with no effect on *Il10* (*p* > 0.05; Table 3). Meanwhile, PZ only increased *Vegfa* mRNA expression, and less potently so than PT (*p* < 0.05; Table 3). Consistent with this data, PT decreased secreted IL-6 (Figure 1), MCP-1, MCP-3, MIP-1α (Figure 2), and leptin (Figure 3), and increased VEGF-A (Figure 3) and adiponectin levels (*p* < 0.05; Figure 1), but did not affect TNF-α (Figure 1) or MIP-1β (*p* > 0.05; Figure 2). In contrast, PZ did not affect the secreted levels of any adipokines compared to LPS + CoCl_2_-stimulation alone (*p* > 0.05; Figure 1, Figure 2 and Figure 3). PPAR-γ inhibition with BADGE partially reversed the angiogenic and anti-inflammatory effects of PT (Table 3) with the exception of *adiponectin* and *Pparγ* mRNA expression and secreted adiponectin and leptin, wherein BADGE totally reversed the effects of PT on adiponectin (*p* < 0.05; Table 3; Figure 1) and had no effect on *Pparγ* mRNA expression (Table 3) or secreted leptin (*p* > 0.05; Figure 3). Further, BADGE totally reversed the effect of PZ on *Vegfa* mRNA expression (*p* < 0.05; Table 3). Finally, the secreted levels of IL-1β and Angptl4 were undetectable in LPS + CoCl_2_-stimulated cultures. Collectively, these data support the anti-inflammatory and angiogenic effects of PT as well as its superior effectiveness compared to PZ in adipocytes under combined inflammatory and hypoxic conditions representative of the obese AT microenvironment. These data also confirm the role of PPAR-γ in mediating these effects, as well as suggest an additional PPAR-γ-independent mechanism of PT action. 

### 3.4. PT but not PZ reduced ROS Accumulation and NF-κB Activation in LPS + CoCl_2_-Stimulated Adipocytes, in Part by a PPAR-γ-Dependent Mechanism

**Figure 4:**
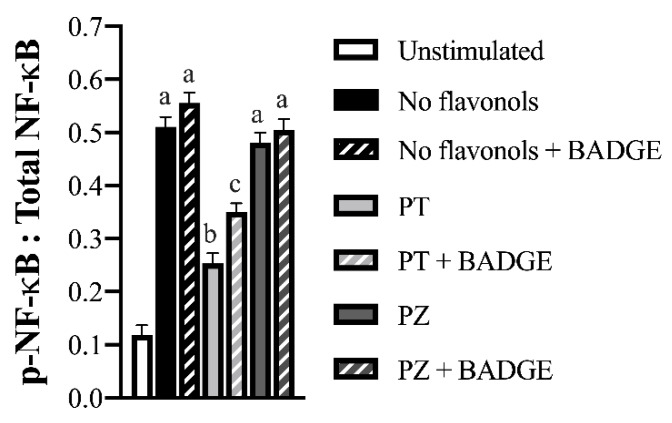
Activated (i.e., phosphorylated) nuclear factor kappa-light-chain-enhancer of activated B cells (NF-κB p65, Ser536): total NF-κB p65 in LPS + CoCl_2_-stimulated adipocytes co-treated with PT or PZ, with or without BADGE. Values are means ± SEM; *n* = 9/treatment. Data were analyzed by two-way ANOVA and means without a common letter differ within cultures treated ± BADGE, *p* < 0.05.

**Figure 5:**
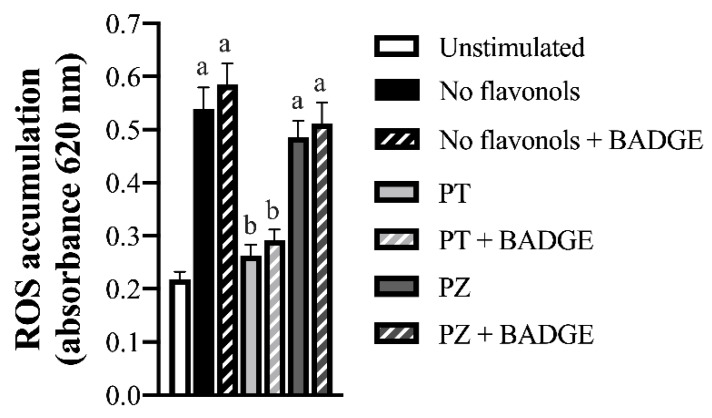
Cellular reactive oxygen species (ROS) accumulation in LPS + CoCl_2_-stimulated adipocytes co-treated with PT or PZ, with or without BADGE. Values are means ± SEM; *n* = 9/treatment. Data were analyzed by two-way ANOVA and means without a common letter differ within cultures treated ± BADGE, *p* < 0.05.

NF-κB p65 activation and ROS accumulation were measured in LPS + CoCl_2_-stimulated cultures to determine additional mechanisms through which PT exerts its angiogenic and anti-inflammatory effects. As expected, LPS + CoCl_2_ stimulation increased the adipocyte ratio of phosphorylated (i.e., activated) to total NF-κB p65 (Ser536) (*p* < 0.05; Figure 4), and increased ROS accumulation compared to the unstimulated condition (*p* < 0.05; Figure 5). Compared to LPS + CoCl_2_-stimulated adipocytes, PT reduced the ratio of phosphorylated to total NF-κB p65 (*p* < 0.05; Figure 4); an effect that was partially reversed by BADGE (*p* < 0.05; Figure 4). Likewise, PT reduced ROS accumulation (*p* < 0.05; Figure 5) and PPAR-γ antagonism with BADGE had no relative effect (*p* > 0.05; Figure 5). In contrast, PZ did not affect the adipocyte ratio of phosphorylated to total NF-κB p65 (Figure 4) or ROS accumulation (Figure 5) compared to the LPS + CoCl_2_-stimulated condition (*p* > 0.05). These data provide mechanistic insight into the anti-inflammatory and angiogenic effects of PT relative to PZ in adipocytes and further support an additional PPAR-γ-independent mechanism of action for PT in a combined inflammatory and hypoxic microenvironment representative of obese AT.

### 3.5. PT but not PZ Modulates Cellular Regulators of Apoptosis in LPS + CoCl_2_-Stimulated Adipocytes, in Part by a PPAR-γ-Dependent Mechanism

**Figure 6:**
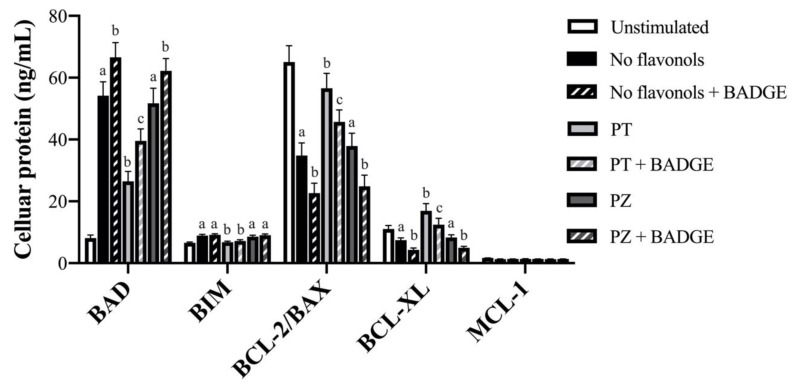
Cellular protein levels of regulators of apoptosis of the B-cell lymphoma 2 (BCL-2) protein family (BCL-2-associated agonist of cell death (BAD), BCL-2-like protein 11 (BIM), BCL-2/BCL-2-like protein 4 (BAX), B-cell lymphoma-extra-large (BCL-XL)) in LPS + CoCl_2_-stimulated adipocytes co-treated with PT or PZ, with or without BADGE. Values are means ± SEM; *n* = 9/treatment. Data were analyzed using a two-way ANOVA and means without a common letter differ within cultures treated ± BADGE, *p* < 0.05.

Cellular regulators of apoptosis of the BCL-2 family were measured in LPS + CoCl_2_-stimulated cultures to determine the effects of PT and PZ on markers of cell survival in an inflammatory and hypoxic microenvironment reflective of the obese AT milieu. As expected, in general, LPS + CoCl_2_ stimulation increased cellular apoptotic and decreased anti-apoptotic protein levels compared to the unstimulated condition (*p* < 0.05; Figure 6). Interestingly, PPAR-γ antagonism with BADGE further increased apoptotic BAD levels and further decreased anti-apoptotic BCL-2/BAX and BCL-XL levels in LPS + CoCl_2_ stimulated-adipocytes (*p* < 0.05; Figure 6), with no effect on apoptotic BIM (*p* > 0.05; Figure 6). Consistent with the anti-inflammatory and angiogenic effects of PT in the LPS + CoCl_2_-stimulated condition, PT reduced BAD and BIM, and increased BCL-2/BAX and BCL-XL (*p* < 0.05; Figure 6). The anti-apoptotic effects of PT on BAD, BCL-2/BAX, and BCL-XL were partially reversed by BADGE (*p* < 0.05; Figure 6), whereas BADGE had no effect on BIM (*p* > 0.05; Figure 6). Finally, PZ did not affect cellular levels of regulators of apoptosis compared to LPS + CoCl_2_-stimulated adipocytes (*p* > 0.05; Figure 6), and anti-apoptotic MCL-1 was only minimally detected in LPS + CoCl_2_-stimulated cultures and was unaffected by treatment (*p* > 0.05; Figure 6). Consistent with its anti-inflammatory and angiogenic effects, these data suggest PT promotes an anti-apoptotic protein profile in adipocytes exposed to combined inflammation and hypoxia, representative of the obese AT microenvironment.

### 3.6. PT and PZ Treatment of LPS + CoCl_2_-Stimulated Adipocytes in Turn Modulate Macrophage Expression of M1 and M2 Polarization Markers

Macrophages were cultured in conditioned media collected from adipocytes stimulated with LPS + CoCl_2_ to determine the effects of (adipocyte treatment with) PT and PZ in modulating mRNA expression of markers of macrophage polarization in response to adipocyte inflammation and hypoxia. As expected, conditioned media collected from LPS + CoCl_2_-stimulated adipocytes in turn increased macrophage mRNA expression of markers of M1 polarization and decreased that of M2 polarization compared to the effects of unstimulated adipocyte conditioned media on macrophages (*p* < 0.05; Table 4). Consistent with our secreted inflammatory (Figure 1) and macrophage chemotactic adipokine (Figure 2) data in LPS + CoCl_2_-stimulated adipocytes, PT (co-treatment of adipocytes) reduced macrophage mRNA expression of the M1 polarization markers *iNos*, *Il6*, and *Tnfα*, and increased expression of the M2 polarization markers *Arg1*, *Il10*, and *Tgfβ* (*p* < 0.05; Table 4). Unexpectedly, despite no effects on secreted adipokines in LPS + CoCl_2_-stimulated adipocytes, PZ (co-treatment of adipocytes) also reduced macrophage mRNA expression of *Il6* and *Tnfα* (*p* < 0.05; Table 4). Interestingly, adipocyte PPAR-γ inhibition with BADGE in turn further increased macrophage mRNA expression of *iNos*, *Il6*, and *Tnfα* (*p* < 0.05; Table 4) but did not affect expression of M2 polarization markers (*p* > 0.05; Table 4). The effects of PT on macrophage mRNA expression of M1 and M2 polarization markers were partially reversed by BADGE (*p* < 0.05; Table 4) with the exception of *Tfgβ* which was unaffected by BADGE (*p* > 0.05; Table 4); whereas, in contrast, the effects of PZ on M1 polarization markers were totally reversed by BADGE (*p* < 0.05; Table 4). These data suggest PT and PZ modulation of the adipocyte response to combined inflammation and hypoxia in turn mitigates macrophage M1 polarization and promotes M2 polarization, and supports the role of PPAR-γ in mediating PT and PZ modulation of adipocyte-macrophage interactions.

## 4. Discussion

Chronic AT inflammation, as an adaptive response to adipocyte hypoxia [9], represents a pivotal target for dietary intervention to mitigate the development of obesity-associated metabolic diseases, such as T2D and CVD [8]. Using culture conditions reflective of the obese AT hypoxic and inflammatory microenvironment, we provide evidence that the apple flavonols PT and PZ reduce adipocyte inflammation and promote the production of angiogenic factors; effects which have been shown to contribute to preserving AT insulin sensitivity [35]. Importantly, the anti-inflammatory and angiogenic effects of PT were consistently more potent than PZ. Further, we showed the effects of PT and PZ were partly or totally mediated through a PPAR-γ-dependent mechanism, with reduced NF-κB activation and ROS accumulation as potential additional interrelated mechanisms through which PT exerts its more potent effects. Finally, the ability of PT to promote angiogenesis and reduce ROS accumulation, a by-product of metabolic stress [6], was supported by altered levels of cellular mediators of apoptosis towards an anti-apoptotic profile. Collectively, our data provide mechanistic insight into the potential for apple-derived flavonols to attenuate critical aspects of the obese AT phenotype, and thus suggest a relatively common dietary component as a useful strategy for preventing chronic metabolic diseases.

To our knowledge, this is the first report of the effects of PT and PZ in LPS-stimulated (mature) adipocytes, and the first report under hypoxic conditions in any cell type. Nonetheless, the PT- and PZ-mediated anti-inflammatory effects observed herein are consistent with other in vitro evidence. In one report of unstimulated adipocytes, treatment with 50 μM PT reduced mRNA expression of IL-6 and MCP-3 and increased mRNA expression of both adiponectin and its receptor [24]. In another report, in TNF-α-stimulated adipocytes, pre-treatment with 10, 30, and 100 μM PT or 100 μM PZ reduced secreted MCP-1 and/or another immune cell chemotactic adipokine, regulated on activation, normal T-cell expressed and secreted [26]. Likewise, in LPS-stimulated macrophages, pre-treatment with 10, 30, and 100 μM PT or 100 μM PZ reduced secreted IL-6 and/or TNF-α levels [25], as did pre-treatment with 10 and 30 μg/mL PT or PZ [36]. Notably, the high concentration of LPS (1000 ng/mL) in these works compared to the obese physiological dose (10 ng/mL) used in our cultures suggests the high anti-inflammatory potency of PT and, to a lesser extent, PZ. Further, the timing of PT and PZ treatment and inflammatory stimulation (with LPS or TNF-α) differed between these studies [25,26,36] and ours; it is possible that PT or PZ pre-treatment in these studies primed the adipocyte/macrophage response to the inflammatory stimuli towards an anti-inflammatory phenotype. Still, similar to the current study design, co-treatment with 45 μM PT or PZ was estimated to be effective at reducing secreted TNF-α levels in (500 ng/mL) LPS-stimulated macrophages [37]. While macrophages are the primary cellular source of TNF-α in AT [38], its mRNA and secreted protein levels were reportedly doubled in adipocytes isolated from obese compared to lean humans [39]. Interestingly, in the current study, TNF-α mRNA and secreted proteins levels were only increased and influenced by PT in the LPS + CoCl_2_-stimulated condition, thus further supporting these aforementioned data on the anti-inflammatory potential of PT and PZ in the complex obese AT microenvironment.

The preventative effects of PT and PZ on obese AT inflammation as supported by the current and aforementioned [24,25,26,36] in vitro work are consistent with the limited in vivo reports [28,29]. To our knowledge, there are no in vivo comparisons of PT and PZ in the context of obesity-associated inflammation and hypoxia. However, it is conceivable that PT would be more effective, as PZ is suggested to be hydrolyzed by gut lactase PZ hydrolase, yielding the PT aglycone for absorption [31]. Indeed, a near equal plasma concentration of total PT was observed in PT- and PZ-fed rodents, whereas PZ was not detected in the plasma of either dietary group [31]. Further, in addition to the C-4′ hydroxyl group which PT and PZ share, and as our results would support, PT may be a more effective PPAR-γ agonist given its free C-2′ hydroxyl group where PZ is instead glycosylated [40], and which is also suggested to participate in stabilizing ROS [41]. PT has also been shown to mitigate ROS via endogenous antioxidant pathways involving nuclear factor erythroid 2-related factor 2, superoxide dismutase, and glutathione [42]. Collectively, these kinetic and structural differences between PT and PZ may explain the PPAR-γ-dependent and -independent inhibitory effects of PT on NF-κB activation, ROS accumulation, and ensuing adipokine regulation, whereas PZ was relatively inert in the LPS + CoCl_2_-stimulated condition.

Another novel aspect of the current study is the addition of CoCl_2_ to adipocyte cultures alone and in combination with LPS to measure the effect of PT and PZ in mediating the hypoxic response. In the obese AT condition of sustained hypoxia, HIF-1 contributes to AT fibrosis instead of angiogenesis, thus restricting adipocyte (and AT) expansion and lipid storage, and inducing inflammation [43] and eventual apoptosis [15]. In the current study, PT increased angiogenic adipokine expression and/or secreted levels, whereas PZ was relatively inert. Interestingly though, PT reduced leptin mRNA expression in all culture conditions, as well as secreted leptin in the LPS + CoCl_2_-stimulated condition, perhaps to mitigate the potential inflammatory effects of leptin despite its angiogenic effects [44], but further study is required. Nonetheless, PT also modulated cellular levels of apoptotic proteins towards an anti-apoptotic profile. Although these data were unsupported by any differences in cell viability (and we did not measure apoptosis directly), these PT-mediated effects may represent an early, protective response to sustained hypoxia in obese AT, though further study is required.

Hypoxic areas of AT are classically co-localized with macrophages, indicating an association between hypoxia and immune cell recruitment and infiltration [45]. Further, under hypoxic conditions, macrophages were observed to polarize to the M1 inflammatory phenotype [46], and were the major source of inflammatory adipokines amongst all AT immune cells [45], whereas anti-inflammatory M2 but not M1 macrophages were reported to contribute to angiogenesis in vivo [47]. In the current study, PT co-treatment of LPS + CoCl_2_-stimulated adipocytes in turn modulated macrophage mRNA expression of polarization markers towards the M2 phenotype, most of which was partially dependent on adipocyte PPAR-γ activity. Unexpectedly, despite no effects on secreted adipokines in LPS + CoCl_2_-stimulated adipocytes, PZ also reduced macrophage IL-6 and TNF-α mRNA expression, which was totally dependent on adipocyte PPAR-γ activity. In addition to reducing inflammatory adipokine production and increasing adiponectin, it is conceivable that PT and PZ activation of adipocyte PPAR-γ also interfered with LPS- and hypoxia-induced lipolysis and instead promoted lipogenesis [48]. Indeed, adipocyte-derived fatty acids have been shown to promote macrophage polarization towards the M1 phenotype [49]. However, further study is required to understand the role of PT and PZ in mediating adipocyte lipogenesis and paracrine interactions with macrophages as controversy exists [26].

Despite our efforts to design cell culture models that are physiologically relevant to obese AT in vivo, it must be acknowledged that dietary flavonols are rarely consumed in isolation, but rather as part of complex food matrices containing fiber, phytosterols, vitamins, minerals, and other molecules that may synergize or antagonize them directly or through secondary signaling pathways. Further, while 100 μM PT (as utilized in the current study) is reportedly representative of the maximum plasma concentration of total PT that was achieved in PT- and PZ-supplemented rats, the majority of plasma PT was conjugated in the form of glucuronides and sulfates [31] which may have health effects separate from PT. Similarly, our timing of LPS and CoCl_2_ stimulation may not adequately reflect the pathology of expanding AT in that AT hypoxia is suggested to precede inflammation in obesity [35]. Finally, the effects of CoCl_2_ on some inflammatory and angiogenic adipokine mRNA expression have been shown to peak at 8–16 h of stimulation, but to be lost at 24 h as employed in the current study; whereas the effects of ambient hypoxia peaked at 24 h [12].

## 5. Conclusions

This study provides evidence for the anti-inflammatory and angiogenic effects of the apple flavonol PT in models designed to mimic the obese AT microenvironment, including inflammation and hypoxia, which are interrelated conditions that drive AT metabolic dysfunction and contribute to T2D and CVD [8,9]. We showed PT consistently mitigates inflammatory adipokines whilst increasing adiponectin and angiogenic adipokine production in LPS-, CoCl_2_-, and LPS + CoCl_2_-stimulated adipocytes. This data was complimented by blunted adipocyte NF-κB activation and ROS accumulation, and alterations in the expression of apoptotic mediators towards an anti-apoptotic profile. In turn, LPS + CoCl_2_-stimulated adipocyte co-treatment with PT mitigated macrophage expression of M1 polarization markers and promoted expression of M2 polarization markers. Finally, we confirmed the effects of PT are partially dependent on PPAR-γ activity and confirmed the superior effectiveness of PT compared to PZ towards these endpoints. Collectively, our findings provide mechanistic support for the consumption of apples, a relatively common dietary component, in the prevention of obesity-associated disease.

## Figures and Tables

**Table 1:** mRNA expression in lipopolysaccharide (LPS)-stimulated adipocytes treated with phloretin (PT) or phlorizin (PZ), with and without bisphenol A diglycidyl ether (BADGE) ^1^.

Gene	No Flavonols	PT	PZ	No Flavonols	PT	PZ
	−BADGE			+BADGE		
Inflammatory adipokines						
Il1β	2.25 ± 0.07 ^a^	1.95 ± 0.07 ^b^	1.98 ± 0.03 ^b^	2.27 ± 0.08 ^a^	2.16 ± 0.04 ^b,^*	2.24 ± 0.05 ^a^
Il6	2.69 ± 0.08 ^a^	0.94 ± 0.06 ^b^	1.25 ± 0.05 ^c^	2.71 ± 0.09	2.58 ± 0.05 *	2.67 ± 0.06 *
Mcp1	5.40 ± 0.16 ^a^	1.89 ± 0.11 ^b^	2.52 ± 0.10 ^c^	5.43 ± 0.18	5.36 ± 0.13 *	5.36 ± 0.13 *
Tnfα	1.06 ± 0.03	0.99 ± 0.03	1.00 ± 0.04	1.01 ± 0.03	1.04 ± 0.05	1.00 ± 0.03
Anti-inflammatory adipokines						
Adiponectin	0.77 ± 0.03 ^a^	1.39 ± 0.02 ^b^	1.21 ± 0.04 ^c^	0.79 ± 0.03	0.84 ± 0.02 *	0.83 ± 0.03 *
Il10	1.06 ± 0.04	1.01 ± 0.02	0.98 ± 0.03	1.01 ± 0.02	1.01 ± 0.03	0.97 ± 0.03
Angiogenic factors						
Vegfa	1.23 ± 0.05	1.27 ± 0.04	1.24 ± 0.04	1.26 ± 0.04	1.26 ± 0.08	1.25 ± 0.05
Angptl4	1.21 ± 0.05	1.20 ± 0.03	1.20 ± 0.02	1.21 ± 0.03	1.21 ± 0.06	1.21 ± 0.04
Leptin	2.56 ± 0.13 ^a^	1.72 ± 0.11 ^b^	2.51 ± 0.13 ^a^	2.93 ± 0.14 *	2.88 ± 0.09 *	2.84 ± 0.05 *
Transcription factors						
Hif1α	1.05 ± 0.03	0.98 ± 0.03	1.00 ± 0.04	1.00 ± 0.03	1.03 ± 0.05	0.99 ± 0.03
Nfκb	1.28 ± 0.07	1.31 ± 0.05	1.36 ± 0.05	1.34 ± 0.07	1.30 ± 0.10	1.38 ± 0.07
Pparγ	1.08 ± 0.04	1.10 ± 0.03	1.03 ± 0.02	1.05 ± 0.03	1.03 ± 0.02	0.99 ± 0.02

^1^ Values are means ± standard error of the mean (SEM); *n* = 9/culture condition. Data were normalized to *Rplp0* mRNA expression and are presented as fold-changes relative to unstimulated adipocytes. Data were analyzed by two-way ANOVA. Means without a common letter differ within cultures treated ±BADGE, *p* < 0.05. * Different from −BADGE, *p* < 0.05.

**Table 2:** mRNA expression in CoCl_2_-stimulated adipocytes treated with PT or PZ, with and without BADGE ^1^.

Gene	No Flavonols	PT	PZ	No Flavonols	PT	PZ
	−BADGE			+BADGE		
Inflammatory adipokines						
Il1β	5.96 ± 0.24 ^a^	2.93 ± 0.09 ^b^	5.39 ± 0.16 ^c^	5.96 ± 0.14 ^a^	3.78 ± 0.06 ^b,^*	5.95 ± 0.13 ^a,^*
Il6	5.03 ± 0.20 ^a^	2.48 ± 0.07 ^b^	3.70 ± 0.11 ^c^	5.03 ± 0.12 ^a^	3.48 ± 0.08 ^b,^*	5.03 ± 0.11 ^a,^*
Mcp1	10.5 ± 0.32 ^a^	4.98 ± 0.15 ^b^	7.42 ± 0.22 ^c^	10.1 ± 0.24 ^a^	6.99 ± 0.16 ^b,^*	10.1 ± 0.21 ^a,^*
Tnfα	1.07 ± 0.03	1.01 ± 0.03	0.99 ± 0.03	1.03 ± 0.02	1.03 ± 0.03	1.00 ± 0.03
Anti-inflammatory adipokines						
Adiponectin	0.56 ± 0.02 ^a^	0.91 ± 0.02 ^b^	0.66 ± 0.02 ^c^	0.58 ± 0.02	0.60 ± 0.02 *	0.57 ± 0.02 *
Il10	0.72 ± 0.02	0.73 ± 0.03	0.69 ± 0.02	0.70 ± 0.03	0.74 ± 0.03	0.72 ± 0.03
Angiogenic factors						
Vegfa	3.25 ± 0.16 ^a^	5.30 ± 0.26 ^b^	3.97 ± 0.16 ^c^	3.33 ± 0.12 ^a^	4.15 ± 0.11 ^b,^*	3.28 ± 0.16 ^a,^*
Angptl4	2.35 ± 0.11 ^a^	4.89 ± 0.13 ^b^	2.30 ± 0.03 ^a^	2.38 ± 0.07 ^a^	3.84 ± 0.07 ^b,^*	2.28 ± 0.06 ^a^
Leptin	3.60 ± 0.18 ^a^	2.71 ± 0.13 ^b^	3.03 ± 0.07 ^c^	4.14 ± 0.10 ^a,^*	3.63 ± 0.15 ^b,^*	3.43 ± 0.15 ^b^*
Transcription factors						
Hif1α	1.07 ± 0.03	1.01 ± 0.03	1.00 ± 0.03	1.03 ± 0.02	1.04 ± 0.03	1.00 ± 0.03
Nfκb	1.62 ± 0.08 ^a^	1.39 ± 0.09 ^b^	1.61 ± 0.06 ^a^	1.84 ± 0.09 *	1.69 ± 0.07 *	1.84 ± 0.10 *
Pparγ	0.60 ± 0.03 ^a^	0.86 ± 0.03 ^b^	0.63 ± 0.03 ^a^	0.64 ± 0.04	0.69 ± 0.02 *	0.63 ± 0.02

^1^ Values are means ± SEM; *n* = 9/culture condition. Data were normalized to *Rplp0* mRNA expression and are presented as fold-changes relative to unstimulated adipocytes. Data were analyzed by two-way ANOVA. Means without a common letter differ within cultures treated ±BADGE, *p* < 0.05. * Different from −BADGE, *p* < 0.05.

**Table 3:** mRNA expression in LPS + CoCl_2_-stimulated adipocytes treated with PT or PZ, with and without BADGE ^1^.

Gene	No Flavonols	PT	PZ	No Flavonols	PT	PZ
	−BADGE			+BADGE		
Inflammatory adipokines						
Il1β	9.04 ± 0.23 ^a^	4.51 ± 0.12 ^b^	8.81 ± 0.17 ^a^	8.97 ± 0.19 ^a^	6.41 ± 0.11 ^b,^*	8.85 ± 0.13 ^a^
Il6	9.07 ± 0.26 ^a^	4.52 ± 0.12 ^b^	8.84 ± 0.17 ^a^	9.00 ± 0.19 ^a^	7.91 ± 0.14 ^b,^*	8.88 ± 0.13 ^a^
Mcp1	22.8 ± 0.50 ^a^	11.2 ± 0.31 ^b^	21.9 ± 0.43 ^a^	22.2 ± 0.5 ^a^	18.4 ± 0.33 ^b,^*	22.0 ± 0.32 ^a^
Tnfα	2.45 ± 0.07 ^a^	1.61 ± 0.04 ^b^	2.42 ± 0.06 ^a^	2.40 ± 0.06 ^a^	2.08 ± 0.06 ^b,^*	2.48 ± 0.06 ^a^
Anti-inflammatory adipokines						
Adiponectin	0.59 ± 0.02 ^a^	0.78 ± 0.02 ^b^	0.55 ± 0.03 ^a^	0.59 ± 0.02	0.57 ± 0.03 *	0.56 ± 0.02
Il10	0.70 ± 0.02	0.73 ± 0.01	0.70 ± 0.03	0.72 ± 0.01	0.71 ± 0.02	0.73 ± 0.03
Angiogenic factors						
Vegfa	1.69 ± 0.08 ^a^	2.39 ± 0.14 ^b^	2.08 ± 0.08 ^c^	1.62 ± 0.08 ^a^	1.90 ± 0.08 ^b,^*	1.61 ± 0.04 ^a,^*
Angptl4	1.92 ± 0.09 ^a^	3.41 ± 0.21 ^b^	1.89 ± 0.06 ^a^	1.84 ± 0.09 ^a^	2.90 ± 0.08 ^b,^*	1.87 ± 0.06 ^a^
Leptin	7.14 ± 0.19 ^a^	4.13 ± 0.10 ^b^	7.10 ± 0.14 ^a^	7.19 ± 0.11 ^a^	5.10 ± 0.16 ^b,^*	7.05 ± 0.11 ^a^
Transcription factors						
Hif1α	2.88 ± 0.08 ^a^	1.90 ± 0.05 ^b^	2.86 ± 0.07 ^a^	2.83 ± 0.07 ^a^	2.45 ± 0.07 ^b,^*	2.92 ± 0.08 ^a^
Nfκb	4.13 ± 0.12 ^a^	2.56 ± 0.13 ^b^	4.08 ± 0.26 ^a^	4.17 ± 0.08 ^a^	3.67 ± 0.11 ^b,^*	4.15 ± 0.10 ^a^
Pparγ	0.50 ± 0.04 ^a^	0.68 ± 0.02 ^b^	0.49 ± 0.01 ^a^	0.45 ± 0.03 ^a^	0.68 ± 0.05 ^b^	0.48 ± 0.04 ^a^

^1^ Values are means ± SEM; *n* = 9/culture condition. Data were normalized to *Rplp0* mRNA expression and are presented as fold-changes relative to unstimulated adipocytes. Data were analyzed by two-way ANOVA. Means without a common letter differ within cultures treated ±BADGE, *p* < 0.05. * Different from −BADGE, *p* < 0.05.

**Table 4:** mRNA expression in macrophages cultured in conditioned media from LPS + CoCl_2_-stimulated adipocytes co-treated with PT or PZ, with and without BADGE ^1^.

Gene	No Flavonols	PT	PZ	No Flavonols	PT	PZ
	−BADGE			+BADGE		
M1 polarization markers						
Cd11b	2.89 ± 0.05	2.73 ± 0.08	2.81 ± 0.13	2.94 ± 0.19	2.93 ± 0.12	2.87 ± 0.12
Cd11c	3.45 ± 0.12	3.41 ± 0.13	3.55 ± 0.12	3.59 ± 0.11	3.50 ± 0.11	3.59 ± 0.10
iNos	10.1 ± 0.27 ^a^	6.49 ± 0.29 ^b^	10.8 ± 0.32 ^a^	13.4 ± 0.61 ^a,^*	10.7 ± 0.46 ^b,^*	12.8 ± 0.39 ^a,^*
Il6	6.16 ± 0.12 ^a^	4.31 ± 0.11 ^b^	5.60 ± 0.08 ^c^	9.46 ± 0.28 ^a,^*	6.53 ± 0.20 ^b,^*	9.20 ± 0.19 ^a,^*
Tnfα	9.10 ± 0.14 ^a^	5.42 ± 0.11 ^b^	7.61 ± 0.17 ^c^	14.9 ± 0.13 ^a,^*	9.18 ± 0.18 ^b,^*	14.6 ± 0.12 ^a,^*
M2 polarization markers						
Cd206	0.96 ± 0.02	1.05 ± 0.04	0.99 ± 0.02	0.98 ± 0.02	1.01 ± 0.03	1.01 ± 0.02
‘Arg1	0.51 ± 0.03 ^a^	0.73 ± 0.01 ^b^	0.57 ± 0.03 ^a^	0.53 ± 0.03 ^a^	0.66 ± 0.03 ^b,^*	0.58 ± 0.02 ^a^
Il10	0.61 ± 0.01 ^a^	0.91 ± 0.02 ^b^	0.64 ± 0.02 ^a^	0.58 ± 0.01 ^a^	0.84 ± 0.02 ^b,^*	0.61 ± 0.01 ^a^
Tgfβ	0.65 ± 0.01 ^a^	0.89 ± 0.02 ^b^	0.68 ± 0.01 ^a^	0.69 ± 0.01 ^a^	0.84 ± 0.03 ^b^	0.71 ± 0.01 ^a^

^1^ Values are means ± SEM; *n* = 9/culture condition. Data were normalized to *Rplp0* mRNA and are presented as fold-changes relative to macrophages cultured in conditioned media collected from unstimulated adipocytes. Data were analyzed by two-way ANOVA. Means without a common letter differ within cultures treated ±BADGE, *p* < 0.05. * Different from −BADGE, *p* < 0.05.

## Supplementary Materials

The following are available online at https://www.mdpi.com/2072-6643/12/5/1386/s1, Table S1: Primer sequences.
