# Supplementary material for: Apple Flavonols Mitigate Adipocyte Inflammation and Promote Angiogenic Factors in LPS- and Cobalt Chloride-Stimulated Adipocytes, in Part by a Peroxisome Proliferator-Activated Receptor-γ-Dependent Mechanism
Source: Nutrients. 2020 May 12;12(5):1386. doi: 10.3390/nu12051386 (PMC7284758; doi:10.3390/nu12051386)
Supplement: Supplementary file 1 [file nutrients-12-01386-s001.pdf]

Table S1. Primer sequences

Gene	Forward primer (5'-3')	Reverse primer (5'-3')
Rplp0	actggtctaggacccgagaag	tcccaccttgtctccagtct
Mcp1	gcctgctgttcacacagttgc	caggtgagtggggcgtta
Il6	aacgatgatgcacttgcaga	gagcattggaaattggggta
Tnfα	catcttctcaaaattcgagtgacaa	tgggagtagacaaggtacaaccc
Ifnγ	atctggaggaactggcaaaa	ttcaagacttcaaagagtctgaggt
Tlr2	ggggcttcacttctctgctt	agcatcctctgagatttgacg
Tlr4	agaaaatgccaggatgatgc	ctgatccatgcattggtaggt
Tnfrsf1a	ggaaagtatgtccattctaagaac	agtcactcaccaagtaggttcctt
Tnfrsf1b	ggctcagatgtgctgtggcta	acaaacatcctgtaatggcttg
Nlrp3	cccttggagacacaggactc	gaggctgcagttgtctaattcc
Caspase1	cccactgctgatagggtgac	gcataggtacataagaatgaactgga
Il1β	agttgacggaccccaaaag	agctggatgctctcatcagg
Il18	caaaccttccaaatcacttcct	tccttgaagttgacgcaaga
iNos	tcctgttgtttctatttcctttgtt	catcaaccagtattatggctcct
Cd11b	agccccacactagcatcaa	tccatgtccacagagcaaag
Cd11c	gagccagaacttcccaactg	tcaggaacacgatgtcttgg
Cd206	ccacagcattgaggagtttg	acagctcatcatttggctca
Arg1	acgccaggaattgttgctat	ccgtgggttcttcacaattt
Il10	ggttgccaagccttatcgga	acctgctccactgccttgct
Tgfβ1	tcagacattcgggaagcagt	acgccaggaattgttgctat
Hif1a	aggctgggaaaagttaggagtg	ggcagcgatgacacagaaac
Nfκb	gagaccggcaactcaagac	ctcaggtccatctccttgggt
Pparγ	tgctgttatgggtgaaactctg	ctgtgtcaaccatggtaatttctt
Adiponectin	caggcatcccaggacatc	tctcacccttaggaccaagaag
Lepin	cagctgcaaggtgcaagaag	gataccgactgcgtgtgtga
Vegfa	ctcggttccagaagtcccat	cactccagggcttcatcgtt
Angptl4	agaaaacatgggctcgaggg	tgggaaccacagttagcacc
